# Structural, Magnetic, Optical and Photocatalytic Properties of Co-Doped ZnO Nanocrystals

**DOI:** 10.3390/ijms26052117

**Published:** 2025-02-27

**Authors:** Manuel Fernando Acosta-Humánez, Claudio J. Magon, Luis Montes-Vides, Juan Jiménez, Ovidio Almanza

**Affiliations:** 1 Departamento de Física, Universidad Nacional de Colombia, Carrera 30 No. 45-03, Bogota 111321, Colombia; mafacostah@unal.edu.co; 2 Instituto de Física de São Carlos, Universidade de São Paulo, São Carlos 13566-590, SP, Brazil; magon@ifsc.usp.br; 3 Departamento de Geociencias, Universidad Nacional de Colombia, Carrera 30 No. 45-03, Edificio Manuel Ancízar, Bogota 111321, Colombia; lamontesv@unal.edu.co; 4 GdS Optronlab, Universidad de Valladolid (UVA), Paseo de Belén 19, 47011 Valladolid, Spain; juanignacio.jimenez@uva.es

**Keywords:** ZnO, electron paramagnetic resonance, raman, photocatalytic activity, nanocrystals

## Abstract

Energy levels associated with several crystalline defects, such as zinc (V_Zn_) and oxygen (V_O_) vacancies, Zn and O interstitials (Zn_i_ and O_i_ respectively), Zn and O antisite defects, and charged oxygen vacancies Vo-, among others, are generated by the introduction of cobalt (Co) into the structure. The effective introduction of Co into the Zn occupancy site was evaluated by XRD and electron paramagnetic resonance. The EPR spectra remain consistent across all doping concentrations of Co^2+^ ions and revealed intriguing features linked to four distinct Co^2+^ paramagnetic centers; among them, a pair of Co^2+^ ions exhibited ferromagnetic coupling. ZnO nanocrystals doped with cobalt were produced by sol gel and their use as photocatalysts were evaluated in the degradation of the Congo red pollutant. The degradation efficiency improved by more than 50% when compared to the efficiency of pure ZnO nanocrystals at the same activity time.

## 1. Introduction

ZnO is considered to be a relatively safe and non-toxic material, but there are concerns about its environmental impact because it can accumulate in soil and water. ZnO has been used for decades in various applications. However, new applications and uses for this versatile material are likely to emerge. Its wide bandgap makes it an attractive material for different applications such as photocatalysis, which uses light energy to activate a catalyst, triggering a chemical reaction. This process has applications in a wide range of fields, including environmental remediation, energy production, and medicine [1].

In photocatalysis, the catalyst is typically a semiconductor material, such as titanium dioxide or zinc oxide, which absorbs photons from the incident light, creating electron–hole pairs. These electrons and holes can then react with molecules adsorbed on the catalyst surface, leading to the formation of new compounds. It is possible to remove harmful substances from the environment without producing any additional waste by using photocatalysts to break down pollutants in air and water. Zinc oxide photocatalysts have been shown to be effective for removing heavy metals from contaminated soils and water [2]. 

Some authors have attributed improvements in ZnO as a catalyst to the presence of energy levels, generated by defects, within the forbidden energy gap [2,3,4]. Zinc (V_Zn_) and oxygen (V_O_) vacancies, Zn and O interstitials (Zn_i_ and O_i_ respectively), Zn and O antisite defects, and charged oxygen vacancies Vo-, among others, can be generated during the process of producing ZnO, or may be introduced by doping with transition metals.

Advances in ZnO synthesis and processing techniques may lead to improved performance and efficiency in environmental science. There are many physical and chemical methods that have been used to produce ZnO or ZnO doped with transition metals. Some of them are thermal evaporation, arc plasma, vapor deposition, sol gel, precipitation, combustion, and hydrothermal techniques, among others. It is known that some electrical and magnetic properties, measured in ZnO, give different values depending on the production method of the material. In the process of doping the ZnO material with transition metals (TM), some studies report that the substitution of the doping metal in the structure is in the place where the Zn is normally found. This statement may not be entirely accurate, and instead it may be possible that the TM is lodged in interstices within the wurtzite-like structure of the ZnO, or in places separate from the exact point where the Zn is in the crystal structure. This may be due to the difference in ionic radii between TM and Zn^2+^ or to the conditions of preparation of the doped samples, e.g., calcination temperature, acidity, etc., which would not allow an isomorphic substitution. Due to the above, some magnetic and optical properties can take on different values, guided by the exact location of the dopant material, within the crystalline structure of ZnO.

The objective of this work was first to evaluate the introduction of cobalt (Co) into the ZnO nanocrystal structure using XRD and EPR. This allowed us to evaluate how effective the doping process is using sol gel as a method to produce nanocrystals. Second, to evaluate the magnetic characteristics of the interactions between neighboring magnetic ions, and third, to evaluate the effective use of cobalt-doped ZnO nanocrystals as a photocatalyst. Its photocatalyst activity was demonstrated with the Congo red contaminant.

## 2. Results

The metal was introduced into the ZnO matrix as much as it was nominally planed. The difference between the real and nominal values was less than 5%.

### 2.1. Scanning Electron Microscopy

**Figure 1:**
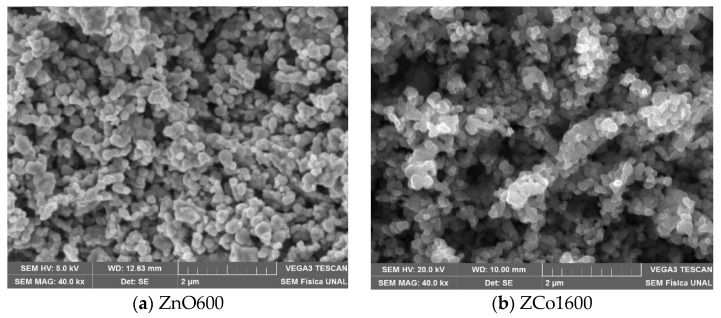

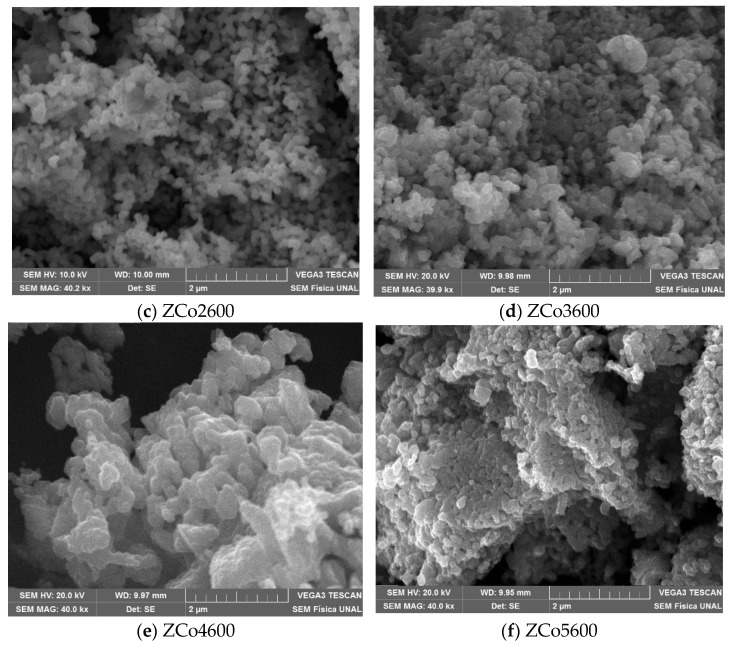
SEM micrographs for (**a**) ZnO sample. (**b**–**f**) sample ZCoX600, with X = 1, 2, 3, 4, and 5.

Figure 1 shows the SEM micrographs obtained from the pure and Co-doped zinc oxide samples. In all of them, the particles exhibit a spherical shape. Particle agglomeration is evident as a result of the calcination temperature used (600 °C). Porosity is also observed in all of them due to the formation of CO_2_, NO_2_, and H_2_O gases during the synthesis process [5,6], as shown in the chemical reaction expressed in Equation (10).

### 2.2. X-Ray Diffraction

**Figure 2:**
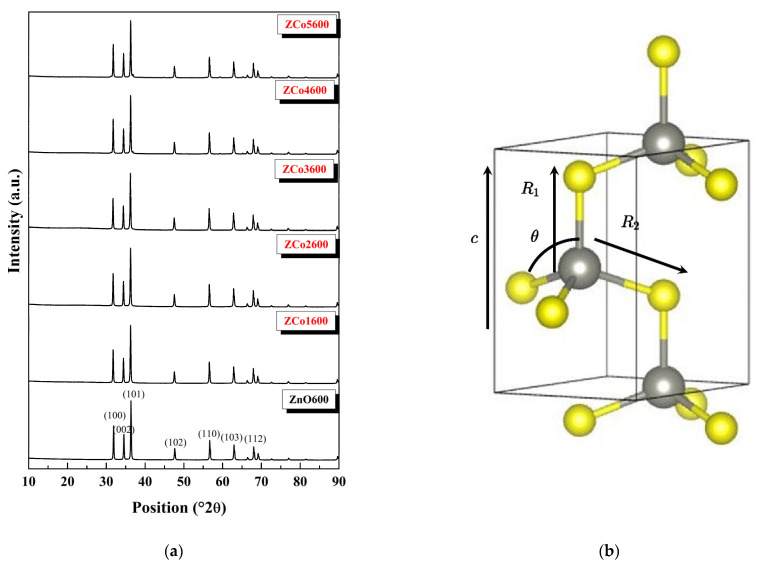
(**a**) X ray diffractogram for cobalt doped ZnO at different Co concentrations. (**b**) shows the wurtzite structure of ZnO and highlights the tetrahedron where the Zn is located or where the dopant metal, Co, would be located.

Figure 2a shows the X-ray diffraction pattern for both the undoped zinc oxide (sample ZnO600) and the same samples doped with cobalt for all of the studied concentrations. The seven most intense diffraction peaks, for all of the samples, correspond to the planes with Miller indices (100), (002), (101), (102), (110), (103) and (112), of the wurtzite-type hexagonal structure of ZnO (PDF-Card 36-1451 (International Centre for Diffraction Data, 2004)) [5]. No peaks associated with other possible structural phases are observed. Figure 2b shows the wurtzite structure of ZnO and highlights the tetrahedron where the Zn is located or where the substitutional dopant metal, Co, would be located in case of having a substitutional doping within the structure. It is an axial symmetry only in cases of having different distances between the metal and the oxygen located in the c direction of the tetrahedron, *R*_1_, and the metal with the neighboring oxygens at the base of the tetrahedron *R*_2_. These values, along with the value of the angle θ, as noted in Figure 1b, were evaluated by X-ray diffraction, using the equations expressed by Böttcher R, et al. [7].

(1) $$R_{1}\left(\delta \right)=uc-\delta$$

(2) R2δ=a23+cu−12−δ2

(3) θδ=arcoscu−12−δR2δ

The parameter u measures the displacement of the Zn atom sublattice with respect to the oxygen sublattice, along the c-axis, and its relationship to the lattice parameters a and c are defined in Equation (4) [8]:

(4) $$u=\left(\frac{1}{3}\right)\left(\frac{a^{2}}{c^{2}}\right)+\frac{1}{4}$$

The average values of the lattice parameters, a and c, for both the doped samples and the pure ZnO sample, are shown in Table 1. The average cell parameters of the all-doped samples did not vary by more than 1% with respect to the cell parameters of the pure ZnO. This slight variation was to be expected considering that the ionic radii of zinc and cobalt in a tetrahedral coordination are very similar (0.60 Å and 0.58 Å, respectively) [9]. The average crystal size depends on the shape and full width at half maximum (FWHM) of the diffraction peaks. In our case, the FWHM was influenced by the inclusion of cobalt atoms in the wurtzite structure of zinc oxide, resulting in a decrease as the amount of Co in the synthesized samples increases. This behavior has already been observed [10]. The mean crystal size, Ds, was evaluated by the Scherrer method. The Ds obtained for the cobalt-doped samples was approximately 32% smaller than Ds the for pure ZnO.

The values defined in the tetrahedron of Figure 2b are also shown in Table 1. The values of *R*_1_ and *R*_2_ of the tetrahedra where Co and Zn are accommodated in the ZnO structure are different. But, the local symmetry around these ions is axial tetrahedral, and this also suggests that Co isomorphically substitutes for Zn in some sites.

### 2.3. Electron Paramagnetic Resonance (EPR)

**Figure 3:**
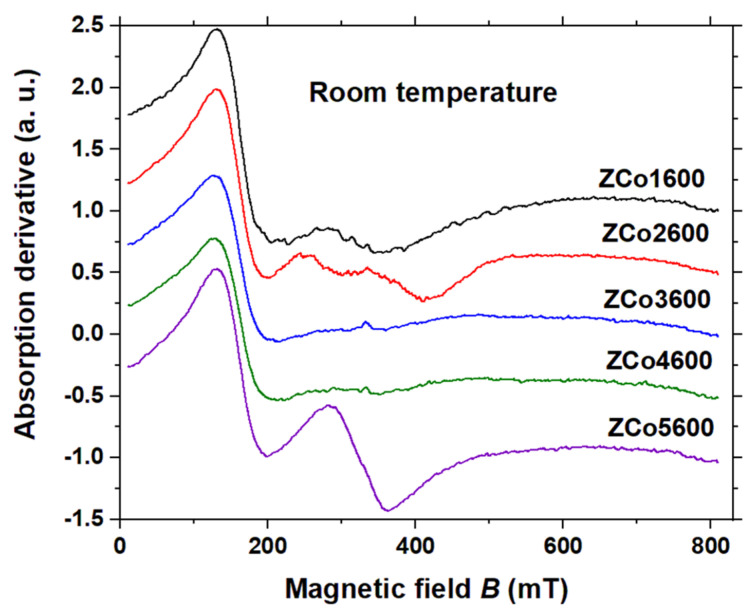
X-band EPR spectra measured at room temperature (295 K) of the sample ZCox600, with x = 1, 2, 3, 4 and 5. Microwave frequency: 9.44 GHz.

The X-band EPR spectra of sample ZCoX600, where X = 1, 2, 3, 4, and 5, acquired at room temperature, are depicted in Figure 3. All of the spectra exhibit similar characteristics, notably marked by significant broadening and structureless spectral features. Despite intriguing disparities among the spectra, no efforts have been made to simulate them based on a comprehensive physical model, which is out of the scope of this work.

Previously observed was a notable rise in linewidth with increasing temperature in analogous samples, a phenomenon elucidated through the delineation of two distinct temperature regimes. Above 60 K, the linewidth escalation correlates with the temperature-dependent spin-lattice relaxation time of Co^2+^ ions. Conversely, below 60 K, the linewidth remains temperature-independent, suggestive of a magnetic origin [11]. To gain a more profound understanding of our findings, it is crucial to undertake a future investigation into the EPR line shape concerning temperature variations specifically for the ZCoX600 samples.

**Figure 4:**
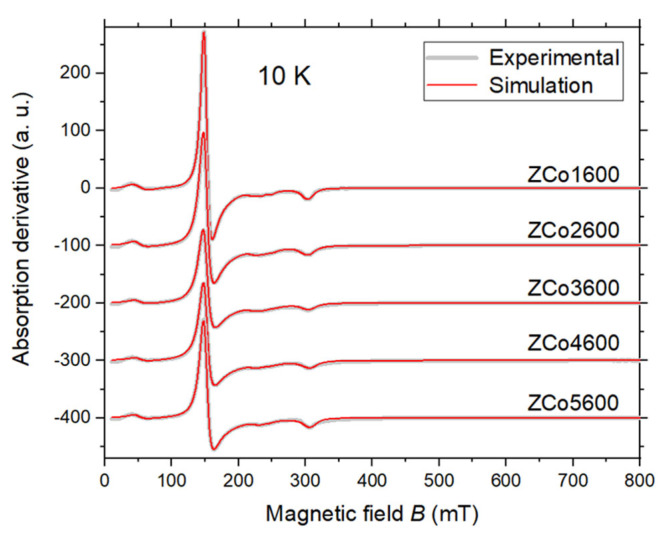
X-band EPR spectra of the sample ZCox600, with x = 1, 2, 3, 4 and 5, measured at 10 K. The spectra are depicted by thick gray traces. The red traces represent the numerical simulation of the experimental data, as elucidated in the accompanying text. Microwave frequency: 9.44 GHz.

Figure 4 illustrates the EPR spectra of the samples acquired at 10 K. While all samples exhibit similar spectra, notable differences in line shapes compared to the room temperature data are apparent: the lines are considerably narrower, and distinct structures indicative of anisotropy in the g-tensor become evident. Upon visual inspection, the spectra reveal three most intense features, with the most prominent at 150 mT and the remaining two at 300 mT and 40 mT, respectively.

**Figure 5:**
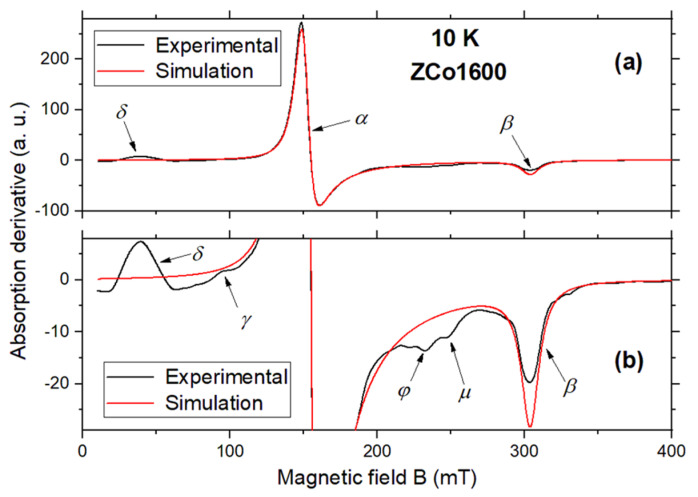
(**a**) X-band EPR spectrum measured at 10 K of the sample ZCo1600. (**b**) shows the magnified spectrum around the baseline. The experimental spectrum is presented in black lines. The numerical simulation, as described in the accompanying text, is presented in red lines. Greek letters denote the main spectral features. Microwave frequency: 9.44 GHz.

To facilitate a clearer examination, the spectrum of sample ZCo1600 is reproduced in Figure 5a, accompanied by a magnified view around the baseline in Figure 5b. Here, the three aforementioned features are labeled as *α*, *β* and *δ*, respectively. Furthermore, the magnification allows for the identification of a minor bump at 95 mT, denoted by *γ*, along with two additional bumps around 235 mT, labeled as *φ* and *μ*. Although other less intense features may be discerned, they were disregarded due to their very weak amplitudes.

Isolated Co^2+^ ions, subjected to an external magnetic field, are usually modeled by the following spin Hamiltonian:

(5) $$H=\beta ({g_{x}B}_{x}S_{x}+{g_{y}B}_{y}S_{y}+{g_{z}B}_{z}S_{z})+D\left[S_{z}^{2}-\frac{1}{3}S(S+1)\right]+E(S_{x}^{2}+S_{y}^{2})$$

In the coordinate system where the g-tensor, $\overset{̿}{g}$, is diagonal, with principal components given by $\left(g_{x},\;g_{y},\;g_{z}\right)$, the first term represents the electronic Zeeman interaction, i.e., the interaction of the electron spin operator, $\vec{S}=(S_{x},S_{y},S_{z})$, with the external applied magnetic field $\vec{B}=(B_{x},B_{y},B_{z})$. *β* is the Bohr magneton and (*D*, *E*) represent the zero-field-splitting tensor for the spins in the system. For the undermentioned simulations, the spin quantum number is always *S* = 3/2 and only axial symmetry will be considered, where *g*_x_ = *g*_y_ = *g*_⊥_ and *g*_z_ = *g*_||_, with *z* being the symmetry axis. Besides, in all cases, *D* ≠ 0 and *E* = 0 were assumed. The ^59^Co nuclei have nuclear spin *I* = 7/2, with 100% natural abundance; therefore, the hyperfine interaction, i.e., the interaction between the electronic and nuclear spins, should manifest as a splitting of each spectral feature into eight lines. However, it is likely that due to dipolar broadening or exchange narrowing processes, the hyperfine structure remains unresolved in the reported single ion spectra. Consequently, this interaction has not been incorporated into the Hamiltonian, Equation (5).

A preliminary analysis of the *α* and *β* features, as indicated in Figure 5, suggests that they can be interpreted as arising from an isolated Co^2+^ ion (*S* = 3/2) with axial symmetry. Indeed, simulating the spectrum by setting *D* = *E* = 0 yields a reasonable agreement with the experimental data, as depicted by the red traces in Figure 4. The least-square fitting of the spectrum provides the g-tensor components: *g*_⊥_ = 4.464 and *g*_||_ = 2.220.

Although there is reasonable agreement between the experimental and simulated spectra, it becomes evident that adjusting the simulated spectrum’s amplitude to fit the intense feature *α* leads to an overestimation of the amplitude of *β* feature. Furthermore, the addition of inhomogeneous broadening does not improve the results.

However, an alternative interpretation of features *α* and *β* is possible. Co^2+^ ions (3d^7^) possess a ground state _4_A^2^(F) and spin *S* = 3/2. The lowest orbital of the free Co^2+^ ion is a singlet, which is four times degenerate. It is well-documented that isolated Co^2+^ ions in ZnO may exhibit a significant axial single-ion anisotropy described by the parameter *D* [12]. An axial distortion of the local crystal field hosting the Co ions contributes to the splitting of this singlet into two Kramers doublets: $\left|S\;,\right.\left.m_{s}\right\rangle$ = $\left|S\;,\right.\left.\pm 3/2\right\rangle$ and $\left|S\;,\right.\left.\pm 1/2\right\rangle$, separated in energy by 2*D*. When 2*D* is comparable to *kT* (where *k* is the Boltzmann constant and *T* is the temperature in Kelvin), and when *E* = 0 (as stated previously), only the fundamental Kramer doublet, $\left|S\;,\right.\left.\pm 1/2\right\rangle$, is preferentially populated. In this scenario, in the presence of an externally applied field, the eigenvalues of the fundamental states are given by [13]:

(6) $$E\approx -D\pm g_{⊥}\;\beta {\;B}_{⊥}\;\text{for}\vec{B}\text{ applied in the perpendicular direction}$$

(7) $$E\approx -D\pm {\frac{1}{2}\;g}_{∥}\;\beta {\;B}_{∥}\;\text{for}\vec{B}\text{ applied in the parallel direction}$$

The approximation holds for *D* much greater than the Zeeman energy. This implies that, in the presence of an external magnetic field, the Zeeman perturbation splits the lower doublet into two axial components. Consequently, the ground state may be treated as an effective spin *S*_eff_ = 1/2 with an associated effective g-tensor given by *g*_⊥eff_ = 2*g*_⊥_ and *g*_||eff_ = *g*_||_. The particular case where *g*_⊥_ ≈ *g*_||_ ≈ 2.2 implies that *g*_⊥eff_ ≈ 4.4 and *g*_||eff_ ≈ 2.2. These effective g-values closely match those determined from the simulation displayed in Figure 5, in which we assumed that *S* = 3/2 and *D* = *E* = 0. It is worth highlighting that substituting *S* = 3/2 with *S*_eff_ = 1/2 does not alter the simulation results.

In this scenario, we propose a second interpretation of the features *α* and *β* as arising from an isolated Co^2+^ (*S* = 3/2), subjected to a significative single-ion anisotropy and possessing an almost isotropic g-tensor. The hypothesis of an effective spin leads to an identical simulation as that depicted in Figure 5. However, as long as *D* remains of the order or greater than *kT*, the simulation is insensitive to the precise value of *D*. To be consistent with our assumptions, a reasonable value was chosen to be *D* = 100 GHz (or, *D* = 4.8 K), which was used for all of the undermentioned simulations.

Upon careful examination of the simulation depicted in Figure 4, it becomes apparent that while the fitting of features *α* and *β* is reasonable, it is not excellent, particularly due to discrepancies in the reproduced amplitude of peak *β*. We hypothesize that this discrepancy could be mitigated by incorporating features *φ* and *μ* into the simulation. Therefore, we now consider that these two additional features represent the parallel transition of two independent Co^2+^ sites in axial symmetry, whose perpendicular transitions coincide with that of feature *α*.

**Figure 6:**
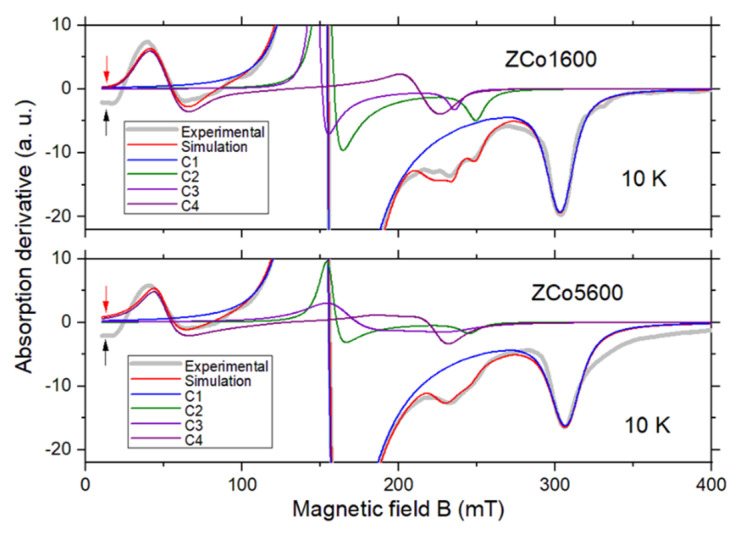
X-band EPR spectrum measured at 10 K of samples ZCo1600 and ZCo5600, with corresponding simulations. For clarity, only a magnified portion of the spectra is shown (for a complete view, see Figure 4). The fitting components C1–C4 are explicitly depicted. The meaning of the arrows is explained in the text. Microwave frequency: 9.44 GHz.

The final simulation of the spectrum of sample ZCo1600, obtained at 10 K, is presented in Figure 4 (in full scale) and in Figure 6 (in magnified scale), with the simulation parameters detailed in Table 2. The multi-component fit comprises four components: one nearly isotropic component (designated as C1 and associated with *β* and part of *α*), two axial components (linked to features *φ*, *μ* and part of *α*, designated as C2 and C3, respectively) and a low field transition (referred to as C4 and associated with features *γ* and *δ*) to be elucidated further.

Now, our focus shifts to features *γ* and *δ*, observed at 40 mT and 95 mT, respectively. The peak detected at 40 mT corresponds to a g-value of approximately 16, a value that is not plausible for a Co^2+^ ion. Similarly, a peak at a low field (around 50 mT) has been noted in analogous samples, but its origin remains poorly understood; possibly it is associated with contamination by impurities [11]. Following this, the aforementioned low field featureParte superior do formulário will be quantitatively investigated by considering a system of two weakly coupled spins with *S* = 3/2, under the influence of easy-plane anisotropy.

The hypothesis concerning the existence of three isolated Co^2+^ ions, in addition to one pair coupled by exchange, can be justified if we consider the quantity of native or induced defects in the crystal due to doping. The existence of these defects will be confirmed in the following sections.

The zero-field spin Hamiltonian for a specific Co^2+^ pair can be formulated as:

(8) $$H=D\left[S_{1z}^{2}-\frac{1}{3}S(S+1)\right]+D\left[S_{2z}^{2}-\frac{1}{3}S(S+1)\right]+J\;{\vec{S}}_{1}\cdot {\vec{S}}_{2}$$

which includes the isotropic Heisenberg coupling.

This Hamiltonian has been rigorously solved for a pair of spins with *S* = 3/2 [14,15]. In our current scenario, we have determined that |*J*| << *D*, which permits us to treat the coupling term (the last term in Equation (8)) as a perturbation. For *J* = 0, the energy level diagram comprises three equidistant levels separated by 2*D*, with the lowest energy level corresponding to spin components of (±1/2; ±1/2). However, the presence of *J* causes the ground state to split into three energy levels described by effective spin quantum numbers *S_eff_* = 0 and *S_eff_* = 1. Besides, the external field introduces additional splitting, resulting in four energy levels due to degeneracy removal.

The simulation of this Hamiltonian, incorporating the Zeeman interaction, is depicted in Figure 4 (in full scale) and in Figure 6 (in magnified scale) and denoted as component C4. The optimal fit was achieved for *J* ≈ −6.3 GHz, indicative of a ferromagnetic interaction. Component C4 accurately accounts for features *γ* and *δ*, along with a minor feature observed at the center of the spectrum (around 220 mT). However, a slight deviation between the experimental and simulated spectra is evident at 10 mT (highlighted by arrows in Figure 6), which remains unexplained. Notably, the negative amplitude of the experimental signal at 10 mT is not attributable to baseline artifacts but rather reflects a genuine absorption signal, as it scales appropriately with the microwave power level.

For simplicity, identical values of *D* and g-tensor components were applied to both ions within the pair. Additionally, several simplifications were implemented in our simulations; for example, the rhombic anisotropy described by *E* was omitted and only the isotropic part of the Heisenberg interaction was considered. It is widely recognized that an excessive number of fitting parameters can compromise the physical relevance of the model. Therefore, rather than pursuing a perfect fitting, our emphasis primarily rested on demonstrating that the pertinent experimental features could be consistently reproduced numerically.

### 2.4. Optical Characterization: Raman, Photoluminescence and Photoreflectance

**Figure 7:**
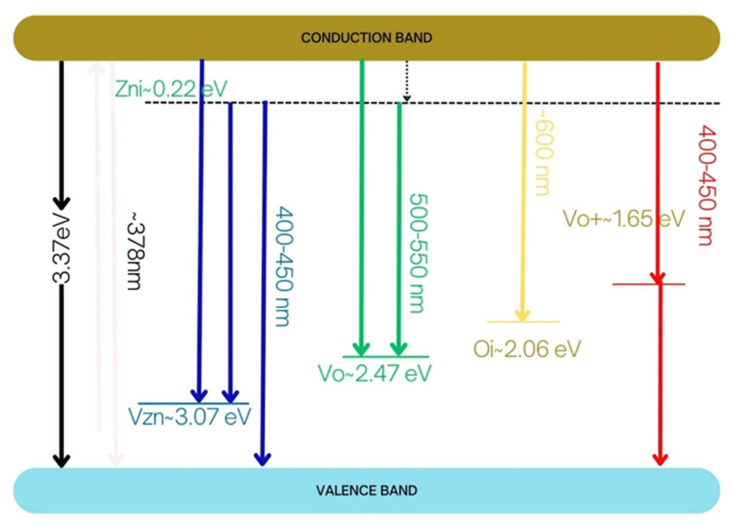
Simplified energy levels of ZnO and the native defects in it. The reported data as described in references [1,2,3,4].

Several types of defects can be found in ZnO. Some of them are native, such as zinc (V_Zn_) and oxygen (V_O_) vacancies, Zn and O interstitials (Zn_i_ and O_i_, respectively), Zn and O antisite defects, and charged oxygen vacancies Vo-, among others. All of them have energy levels located within the bandgap of the parent material, ZnO. Figure 7 shows the energy levels associated with these defects, as calculated by several authors [1,2,3,4]. When transitions occur from the conduction band to the valence band of ZnO, the transition energy is in the ultraviolet, ≈3.27 eV, equivalent to the ZnO bandgap. Zn_i_ defects are located 0.22 eV below the conduction band. The possible transitions between the different levels are shown in Figure 7. Therefore, it is necessary to combine different defects in ZnO in order to have a photocatalyst activated by several energies within the visible region of the spectrum.

**Figure 8:**
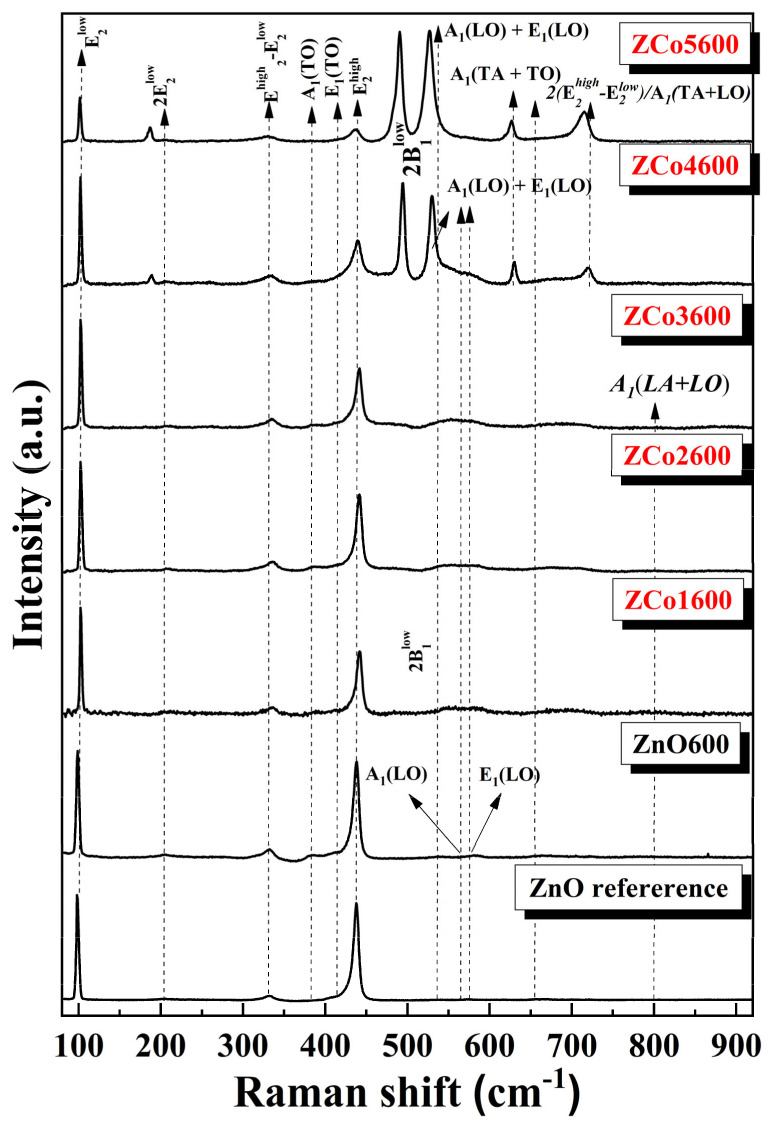
Raman spectrum of cobalt doped ZnO for different Co concentrations.

**Figure 9:**
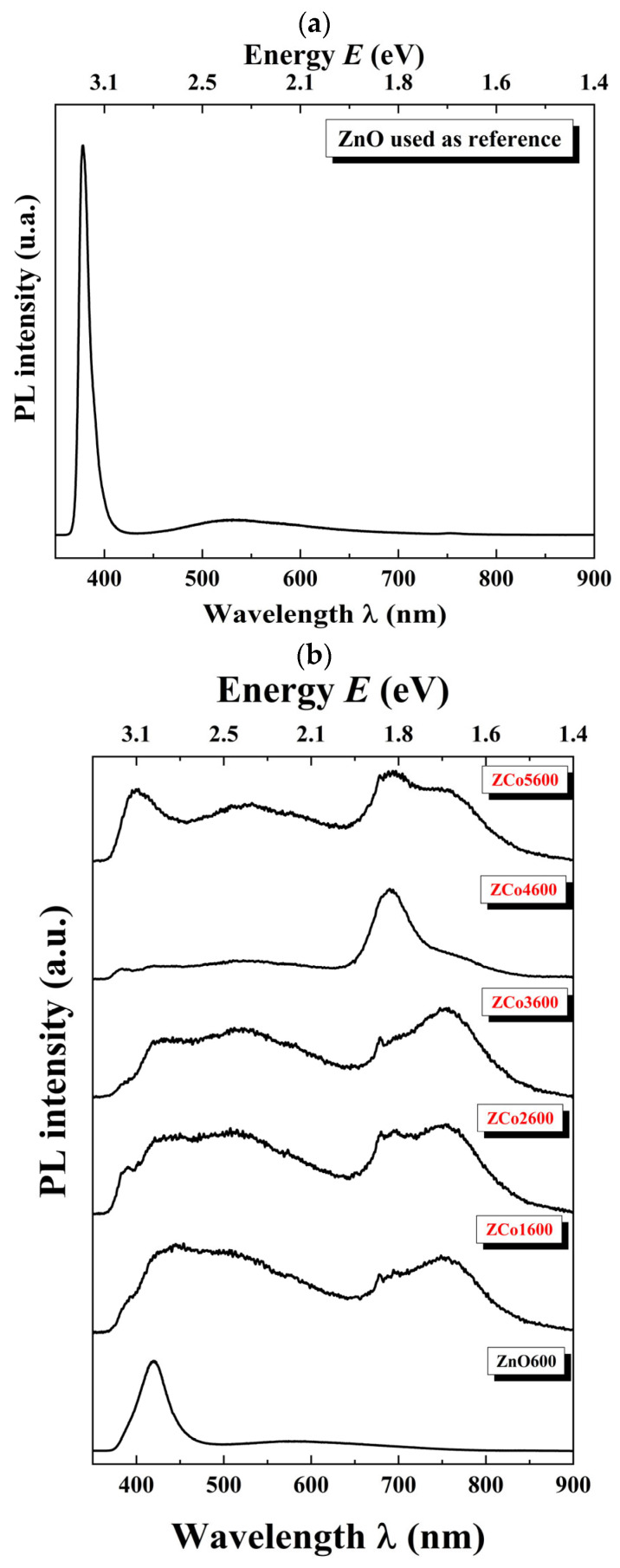
(**a**) Photoluminescence spectrum of pure ZnO. (**b**) Photoluminescence spectrum of Co-doped ZnO samples at different concentrations.

Raman spectra for the calcined materials, for different dopant cobalt concentrations and the reference ZnO sample, are shown in Figure 8. The wurtzite ZnO has the irreducible representation Γ_opt_ = A_1_ + E_1_ + 2E_2_ + 2B_1_; the B_1_ modes are Raman silent, while the A_1_ y E_1_ modes are polar and the E_2_ modes are non-polar and Raman active. The low frequency E_2_^low^ mode mainly involves Zn motion, while E_2_^high^ is related to oxygen motion. The A_1_ modes can hardly be detected for excitation wavelengths above 400 nm. The Raman spectrum recorded under our experimental conditions in the ZnO reference is dominated by the non-polar modes E_2_^low^ (99 cm^−1^) and E_2_^high^ (437 cm^−1^), with a weak contribution of a peak at 333 cm^−1^, which can be associated with an E_2_^high^-E_2_^low^ overtone. The incorporation of Co produces changes in the spectrum: one observes a progressive intensity decrease and broadening of the E_2_^high^ zone center mode, which suggests lattice symmetry breakdown affecting the oxygen sublattice. The E_2_^low^ mode is more stable, and one observes a significant intensity decrease for the highest Co concentration. Significant spectral changes are observed for samples ZCo4600 and ZCo5600, where several overtones are activated by the lattice distortion introduced by the presence of a significant incorporation of Co. For a description of the main modes present in ZnO, see Cuscó et al. [16]. One should pay special attention to the peaks 530 cm^−1^ and 723 cm^−1^, which have been related to 2B_1_^low^ and LA-TO with A_1_ symmetry overtones, respectively [16]; however, they have been also related to local vibration modes (LVMs) associated with cobalt, in particular a complex of cobalt with a donor for the 530 cm^−1^ peak [17] and to substitutional Co in the ZnO lattice [18]. There are two additional bands located at ≈ 490 cm^−1^ and 540 cm^−1^, associated with lattice defects: oxygen vacancies (V_O_) and zinc interstices (Zn_i_), respectively, which have their energy levels within the ZnO bandgap, according to Samadi et al. [4] (see Figure 8 and Figure 9).

Figure 9a shows the photoluminescence spectrum of pure ZnO. The PL peak, at approximately 380 nm, is associated with CB to VB transitions. The PL spectra of Co-doped ZnO samples are shown in Figure 9b. The most prominent peaks in the PL spectra of these samples are above 400 nm and are associated with transitions between the different electronic energy levels generated by defects such as oxygen vacancies (Vo), zinc vacancies (V_Zn_), oxygen interstices (O_i_), and zinc interstices (Zn_i_); see Figure 7. The peak at approximately 408 nm of the 5% Co doped ZnO sample is likely due to transitions between the energy levels associated with Zn_i_ and the valence band or the level close to the VB associated with zinc vacancies. These transitions are marked in blue in Figure 7, because the energy of the transitions is in the blue window of the electromagnetic spectrum. The other large peak shown in the 5% Co doped ZnO sample, located at approximately 695 nm, is associated with transitions between the conduction band and the energy level associated with O_i_. Both defects, Zn_i_ and O_i_, were revealed as major defects in the Raman spectra [19].

**Figure 10:**
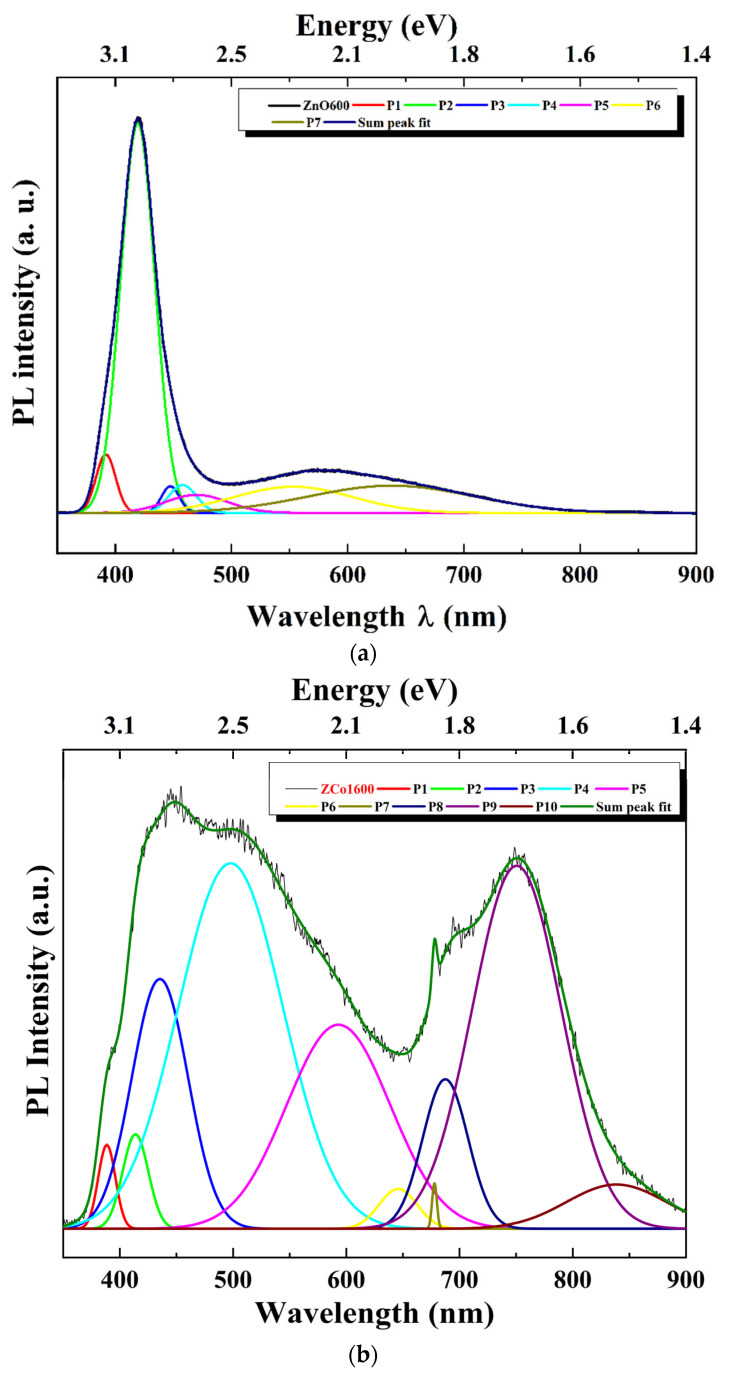
PL deconvolution fit. (**a**) ZnO sample. (**b**) Co-doped ZnO sample at 1% at. (ZCo1600).

In order to further study the peaks observed with this spectroscopic technique, a Gaussian deconvolution adjustment was performed on each PL spectrum, as shown in Figure 10 for the zinc oxide sample (Figure 10a) and the 1% at. Co-doped ZnO sample (ZCo1600, Figure 10b). The results obtained from the adjustment are recorded in Table 3.

It is worth noting that for the synthesized samples, all of the electronic transitions observed in the spectra correspond to those associated with ZnO [2,20]. In all of the samples, it is observed that the presence of visible PL emissions increases with the incorporation of cobalt, compared to those observed in the zinc oxide sample, ZnO600. As the doping molar ratio x increases, different types of defects are generated in the material, leading to various energy sublevels within the ZnO gap associated with them. This results in different emissions toward these sublevels, which are in the visible region of the spectrum. The strong green emission and the appearance of red emissions are typical, resulting from transitions from the conduction band to energy levels associated with zinc and oxygen vacancies, zinc and oxygen interstitials (Zni and Oi), as well as oxygen antisites, or between them.

**Figure 11:**
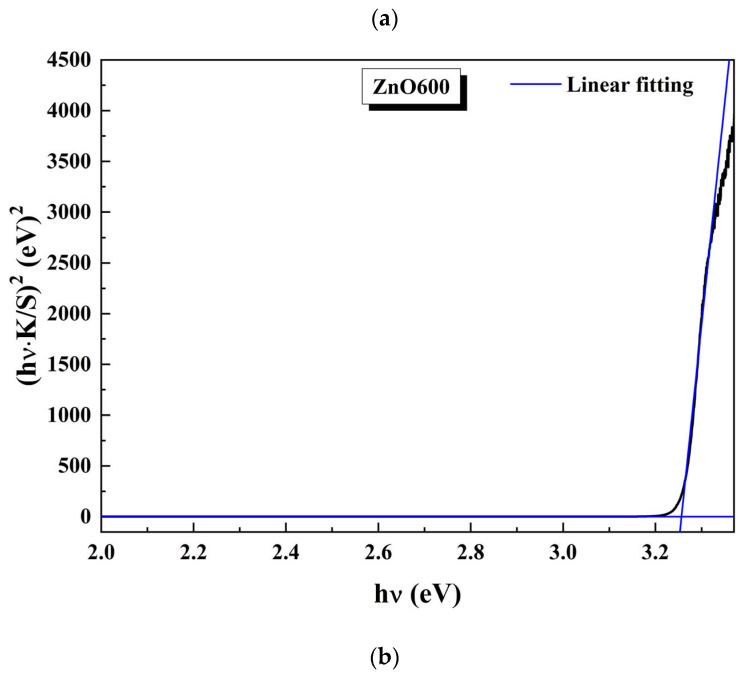

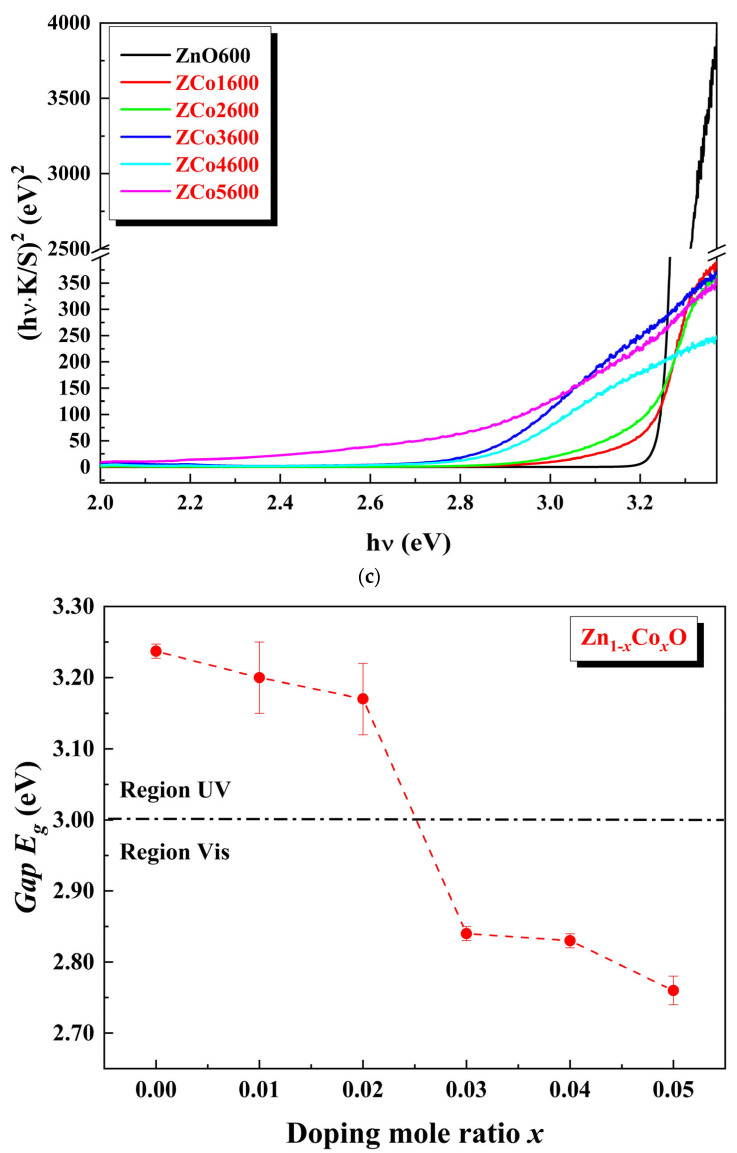
(**a**) Kubelka–Munk (K-M) for ZnO pure. (**b**) Kubelka–Munk (K-M) for Co-doped ZnO. From 1 to 5% at. (**c**) Variation of the gap (•) of Co doped ZnO as a function of the doping molar ratio x for the metals studied.

Figure 11a,b show the modified Tau plot curves of the pure ZnO samples and cobalt doped samples. They were obtained from reflectance spectra measured in the range 350–800 nm and modified by using the Kubelka–Munk equation [21,22], Equation (9), plotting (*k/s∙hv*)^2^ as a function of *hv*. The bandgap is determined by extrapolating the linear portion on the plot when *k = 0:*

(9) $${\left(h\nu \cdot K/S\right)}^{2}=C\left(E_{g}-h\nu \right)$$

Figure 11c shows the variations of the bandgap, E_g_, for the samples studied, as a function of the cobalt concentration. As can be seen, when the Co concentration is higher than 3% at., the effective bandgap in these doped samples is smaller than the bandgap for the ZnO reference sample (3.237 eV). The progressive decrease of the Eg value with the amount of Co introduced results in a significant gap shift toward the visible region of the spectrum; that is, the samples go from absorbing significantly in the ultraviolet (UV) to absorbing in the visible. The samples with the highest Co concentrations, ZCo4600 and ZCo5600, have the smallest effective bandgaps, because of the higher concentration of defects.

## 3. Discussion

**Figure 12:**
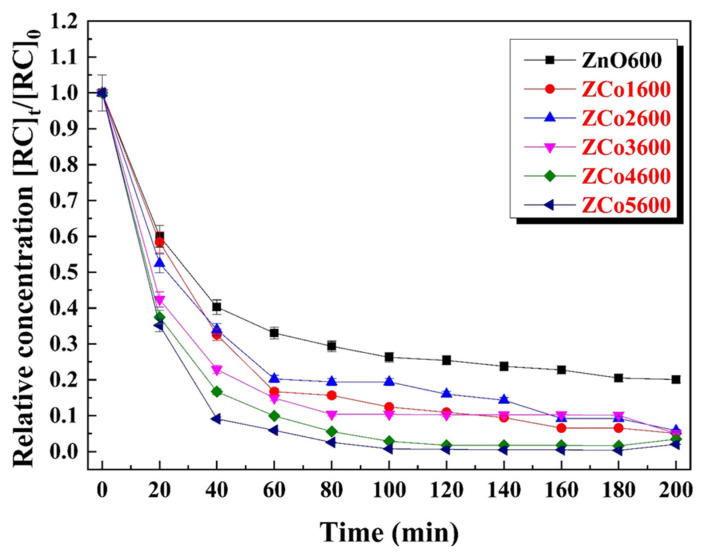
Plots of relative [RC]t/[RC]0 concentrations as a function of time for pure ZnO (black dots) and cobalt doped ZnO.

The degradation, consumption or transformation curves of Congo red (CR) dye, in the presence of semiconducting materials, pure ZnO and Co doped ZnO, at different concentrations, are shown in Figure 12. The relative concentration of Congo red [RC]_t_/[RC]_0_ decreased by 60%, approximately, in a reaction time of 40 min for the solution of CR with pure ZnO nanocrystals, the black dots in Figure 10. That is, only about 40% of the initial concentration of RC remains in the solution, while 60% has been degraded. In this same solution, 100 min after starting the degradation, the relative concentration of Congo Red has decreased to approximately 30% of the initial concentration and no appreciable degradation occurs for longer times. In other words, ZnO in the presence of ultraviolet light degrades 70% of the CR after 100 min of reaction. In the RC reactions with Co doped ZnO, at doping levels of 1, 2, 3, 4 and 5% at., the degradation of the Congo red dye is between 60 and 90% of the initial concentration, depending on the metal concentration in the photocatalyst (see Figure 10), 40 min after starting the reaction. In other words, there is an appreciable improvement in the degradation of this dye when the catalyst is ZnO doped with cobalt. The sample with a doping molar concentration of 5% at. is the most efficient as a photocatalyst, degrading approximately 90% of the RC 40 min after the degradation has started. At 100 min after the start of the reaction, the ZnO samples doped with 4 and 5% Co have degraded practically all of the RC (more than 98% of the initial concentration). This is an important achievement, meaning that a CR solution with the 4 and 5% Co doped ZnO nanocrystals was fully cleaned.

It is true that both the surface area and morphology of nanocrystals can influence the degradation rate of the pollutant agent. However, for all of the materials studied, the shapes of the nanocrystals are hexagonal, regardless of the cobalt concentration in the sample. This was confirmed by TEM. The crystal size was measured for the nanocrystals in the doped samples. The smallest average size was found in the samples doped with cobalt, from 1 to 3% at., but these samples do not exhibit the highest photocatalytic activity. Therefore, we believe that the main contribution to the improvement in degradation comes from the concentration of defects in the doped materials.

## 4. Materials and Methods

The samples were produced by the sol gel method, citrates route, as it is a technique presenting a good control stoichiometry of the synthesized materials. The reagents used were hexahydrated cobalt nitrate (Co(NO_3_)_2_∙6H_2_O, 98% purity, hexahydrated zinc nitrate (Zn(NO_3_)_2_∙6H_2_O, 98% purity, Panreac, Bogotá, Colombia), and monohydrated citric acid (C_6_H_8_O_7_∙H_2_O, 99.5–100.5%, Merck, Bogotá, Colombia). (All the products were purchased in Bogotá, Colombia, from the authorized distributors of these companies).

Stoichiometric quantities of these reagents were diluted in 50 mL of deionized water, forming the corresponding ions at 70 °C under constant magnetic stirring. The citrate/nitrate ratio, c/n, is 1.0 [5]. When Zn^2+^ and Co^2+^ ions are added to the citric acid solution, the formation of zinc citrate and dopant metal occurs. These precursors (zinc, dopant metal and citrate) are calcined at Tc = 600 °C for 12 h, resulting in the formation of cobalt-doped ZnO. The target concentration of cobalt in ZnO was selected from 1 to 5% at. The reaction can be written as:

(10) $$\left(1-x\right)\left\{\left[Zn{\left(H_{2}O\right)}_{6}\right]{\left(NO_{3}\right)}_{2\left(ac\right)}\right\}+x\left\{\left[M{\left(H_{2}O\right)}_{6}\right]{\left(NO_{3}\right)}_{2\left(ac\right)}\right\}+C_{6}H_{8}O_{7}\cdot H_{2}O_{\left(ac\right)}\;+4O_{2}^{*}⟶Zn_{1-x}M_{x}O_{\left(s\right)}+2NO_{2\left(g\right)}+6CO_{2\left(g\right)}+11H_{2}O_{\left(l,g\right)}$$

The samples were labeled as ZCoX600, Z indicating that it is a ZnO sample, CoX600 that it is cobalt doped at concentration X (X = 1, 2, 3, 3, 4 and 5% at.) and calcined at 600 °C.

The chemical analysis of the synthesized samples was performed by atomic absorption (AAS) with Thermo Scientific Solar S equipment (Thermo Scientific, Bogotá, Colombia), operating at wavelengths of 213.9 nm. The morphology of the samples studied was analyzed using a Tesca Vega 3 SB SEM microscope (Tesca, Bogotá, Colombia), operated at a voltage of 30 kV and high pressure. It was not necessary to gold (Au) coat the samples. The micrographs were taken at various depths in secondary electron mode. X-ray diffraction was carried out with a Panalytical X’ Pert Pro MPD diffractometer (Malvern Panalytical, Bogotá, Colombia). All of the diffractograms were measured between 10 and 90° 2θ with step size 0.0260 and copper as the anode. Phase identification was performed by X’ Pert HighScore Plus 2.2 software (Philips Analytical B.V., Almelo, The Netherlands, 2005), considering the ICDD PDF-2 2004 database (International Centre for Diffraction Data, Newtown Square, PA, USA, 2004). The lattice parameters were determined by the Rietveld method and the FULLPROF program. Continuous-wave (CW) EPR spectra were obtained at room temperature and at 10 K, in the range of 10–810 mT, with a Bruker Elexsys E580 spectrometer (Bruker, Bogotá, Colombia) operating at X-band (≈9.5 GHz), equipped with a rectangular cavity Bruker ER4102ST (Bruker, Bogotá, Colombia), a continuous flow liquid helium cryogenic system Oxford ESR900 (Oxford Instruments, Bogotá, Colombia), and a temperature controller Oxford ITC503 (Oxford Instruments, Bogotá, Colombia). The sample was placed at the bottom of a quartz tube (3 mm inside diameter) and inserted in a cryostat at the center of the magnet cavity. To avoid saturation effects, the microwave power was always attenuated at least 6 dB below the saturation threshold. Overmodulation and passage effects were avoided by setting the modulation amplitude and time constant to at least one tenth of the narrowest linewidth and shortest transit time, respectively. Powder solid state EPR simulations were carried out using the functions pepper and esfit of the EasySpin toolbox 5.2.35 of MATLAB-2024 [23]. 

Some of the main simulation parameters will now be briefly described. Besides the spin Hamiltonian parameters, the broadening parameters account for isotropic and anisotropic broadening. The parameter describing isotropic broadening is named lwpp and is used for convolution of the spectrum with Gaussian and/or Lorentzian lineshapes, denoted by lwppG and lwppL, respectively. Peak-to-peak, pp, refers to the field distance between the maximum and the minimum of a first-derivative lineshape (in units of mT). Also, in this work, anisotropic broadening was used to consider distributions of the scalar parameters g_⊥_ and g_||_, specified in EasySpin by dimensionless array gStrain and here denoted as Δg_⊥_ and Δg_||_, respectively. Both parameters represent the Full-Width-at-Half-Maximum, FWHM, of the corresponding distributions, which are treated as uncorrelated. EasySpin also allows us to set the temperature of the experiment, in which case the populations are computed for all energy levels assuming thermal equilibrium and the population differences between two levels are included in the line intensities of the corresponding EPR transition. Additionally, for a multi-component mixture, each component of the spin system has a parameter named *weight*, here denoted by *w*, which specifies the contribution of the corresponding component to the final spectrum. Actually, *w* is proportional to the number of spins in the corresponding component. 

Raman spectra were measured in a LabRam UV-HR 800 Horiba–Jovin Yvon LabRam UV–HR spectrometer (Horiba, Bogotá, Colombia) in micro-Raman measurement mode. Excitation of the sample was achieved by a frequency doubled Nd:YAG laser (532 nm). The measurement range was 80–920 cm^−1^. UV–Vis measurements were performed in a Varian Cary 5000 UV–Vis spectrophotometer (Agilent, Bogotá, Colombia). Measurements were made with a step size of 0.1 nm and a measurement time of 0.1 s in a 350–800 nm spectral window and measurement speed 60 nm/min. Photoluminescence spectra were recorded in the LabRam UV–HR 800 Horiba–Jovin Yvon spectrometer (Horiba, Bogotá, Colombia) in PL measurement mode. The excitation was achieved with a He-Cd UV laser (325 nm). The measurement range was 200–1050 nm.

The photocatalytic activity on the organic dye was adapted from Elaziouti et al. [24] The dye reagent was Congo red (C_32_H_22_N_6_Na_2_O_6_S_2_, 98%, Panreac). A fixed amount of the doped material was used as a catalyst (0.50 g/L), as well as a fixed concentration of Congo red solution (20 mg/L). The pH of the resulting suspension was adjusted to a value of 8.0 with dilute solutions of sodium hydroxide and sulfuric acid (NaOH/H_2_SO_4_) in order to avoid aggregation of the dye [24]. As a radiation source, an 8.0 W UV lamp with emission at 365 nm was used at a fixed distance of 3 cm between the lamp and the suspension. The absorbance of Congo red in the supernatant liquid was measured in a UV–Vis 6320D Jenway spectrophotometer (VWR, Bogotá, Colombia). The wavelength used was the maximum wavelength measured for the dye, λ_max_ = 490 nm.

## 5. Conclusions

Samples of pure ZnO and ZnO doped with cobalt at concentrations ranging from 1 to 5% with an average nanometric crystal size and wurtzite-type structure have been produced by sol gel. Co^2+^ replaces Zn^2+^ in the ZnO lattice, as both ions have approximately the same radius (0.58 Å vs. 0.60 Å). The local symmetry once Co is accommodated in the ZnO lattice does not undergo deformation, preserving its undeformed axial symmetry.

The EPR spectra of all samples, taken at 10 K, were effectively interpreted by considering four distinct paramagnetic centers, each experiencing significant single-ion anisotropy (*D* = 100 GHz). These centers include one comprising a Co^2+^ ion with a nearly isotropic g-tensor, two Co^2+^ ions exhibiting axial symmetry, and a fourth center attributed to two ferromagnetically coupled Co^2+^ ions (*J* = −6368 MHz). Notably, the inclusion of this fourth component provided the first comprehensive explanation for the observed EPR peak at low field, approximately 40 mT.

The cobalt-doped ZnO samples showed abundant crystalline defects, namely oxygen vacancies and zinc interstitials, among others. These defects introduce energy levels located within the ZnO bandgap and reduce the effective bandgap of the doped material by more than 14% at a 5% cobalt concentration. The doped ZnO nanocrystals are a much more effective material than pure ZnO for photo-catalysis, improving by up to 50% their effectiveness in the degradation of the Congo red pollutant. In 60 min, 5% Co doped ZnO degraded more than 90% of the Congo red dye, while pure ZnO only degraded 60%.

## Figures and Tables

**Table 1:** Mean crystal size and mean cell parameters obtained for pure and Co-doped ZnO nanocrystals. X means any Co concentration.

Name	Ds (nm)	a (Å)	c (Å)	*R*_1_(δ) Å	*R*_2_(δ) Å	θ(δ) °
ZnO600	33.7	3.2516	5.2093	1.954	1.997	109.1
ZnCoX60	23.0	3.2523	5.2067	1.969	1.982	108.7

**Table 2:** Parameters obtained from the multi-component simulation of the experimental spectra of samples ZCox600, with x = 1, 2, 3, 4 and 5, taken at 10 K. The meaning of all symbols is explained in the text. Constant parameters not included in the table, and applicable to all components and concentrations, are as follows: *D* = 100 GHz, lwppG = 0, g*S* = 3/2 for components C1–C3 and *S* = 3/2 ⊗ 3/2 for component C4.

	Sample	g_⊥_	g_||_	lwppL (mT)	Δg_||_	*J* (GHz)	*w*
C1	1600	2.236	2.222	7.87	0.085	0	1
	2600	2.237	2.222	10.3	0.10	0	1
	3600	2.240	2.213	11.3	0.095	0	1
	4600	2.235	2.200	11.0	0.095	0	1
	5600	2.239	2.198	10.0	0.080	0	1
C2	1600	2.173	2.703	7.87	0	0	0.105
	2600	2.210	2.686	13.6	0	0	0.180
	3600	2.253	2.650	20.0	0	0	0.289
	4600	2.194	2.709	15	0	0	0.104
	5600	2.154	2.749	8.5	0	0	0.038
C3	1600	2.273	2.859	5.92	0	0	0.047
	2600	2.251	2.844	12.8	0	0	0.088
	3600	2.196	2.845	12.5	0	0	0.151
	4600	2.231	2.841	36.0	0	0	0.193
	5600	2.081	2.912	27.0	0	0	0.065
C4	1600	2.033	2.676	15.9	0.200	−6.37	0.035
	2600	2.009	2.932	16.9	0	−6.37	0.074
	3600	2.035	2.894	17.7	0	−6.33	0.073
	4600	1.996	2.917	17.0	0	−6.35	0.059
	5600	1.963	2.891	16.0	0	−6.40	0.055

**Table 3:** Photoluminescence peaks obtained by Gaussian deconvolution for all samples synthesized.

Sample	Peak (P)	λ (nm)	E (eV)
ZnO600	1	392	3.16
	2	419	2.96
	3	447	2.77
	4	458	2.71
	5	470	2.64
	6	553	2.24
	7	638	1.94
ZCo1600	1	388	3.19
	2	414	3.00
	3	435	2.85
	4	498	2.49
	5	593	2.09
	6	646	1.92
	7	678	1.83
	8	687	1.80
	9	751	1.65
	10	839	1.48
ZCo2600	1	386	3.21
	2	412	3.01
	3	440	2.82
	4	497	2.50
	5	577	2.15
	6	683	1.82
	7	704	1.76
	8	749	1.66
ZCo3600	1	425	2.92
	2	463	2.68
	3	504	2.46
	4	574	2.16
	5	684	1.81
	6	752	1.65
ZCo4600	1	384	3.23
	2	421	2.95
	3	530	2.34
	4	689	1.80
	5	730	1.70
ZCo5600	1	391	3.17
	2	408	3.04
	3	433	2.86
	4	502	2.47
	5	585	2.12
	6	687	1.80
	7	745	1.67

## Data Availability

The raw/processed data required to reproduce these findings cannot be shared at this time due to technical or time limitations.
